# Case report: Dopamine Dysregulation Syndrome, mania, and compulsive buying in a patient with Parkinson's disease

**DOI:** 10.3389/fneur.2023.1290653

**Published:** 2023-11-20

**Authors:** Carlos Silva, Marta Rebelo, Inês Chendo

**Affiliations:** ^1^Psychiatry Department, Department of Neurosciences, Hospital de Santa Maria, Lisbon, Portugal; ^2^Clínica Universitária de Psiquiatria e Psicologia Médica, Faculdade de Medicina, Universidade de Lisboa, Lisbon, Portugal; ^3^Laboratory of Clinical Pharmacology and Therapeutics, Faculdade de Medicina, Universidade de Lisboa, Lisbon, Portugal

**Keywords:** Parkinson's disease, dopaminergic Dysregulation Syndrome, mania, compulsive buying, impulsive-compulsive behaviors

## Abstract

Neuropsychiatric symptoms and syndromes are among the most common non-motor symptoms of Parkinson's Disease but they are frequently unrecognized and untreated. Dopamine Dysregulation Syndrome is an uncommon complication of the treatment of Parkinson's disease, characterized by an addictive use of dopamine far more than the dosage required for treatment of objective motor impairment, leading to severe dyskinesia, euphoria, aggressivity, or psychosis. We present a paradigmatic case of Dopamine Dysregulation Syndrome, Mania, and Compulsive Buying in a 55-year-old male with Parkinson's Disease. We also reviewed the risk factors and the therapeutic management of Dopamine Dysregulation Syndrome in Parkinson's Disease.

## Introduction

Dopamine Dysregulation Syndrome (DDS) is an addictive pattern of dopamine replacement therapy use, above the prescribed dosage and those required to control motor symptoms (1–3). This therapeutic abuse results in severe motor dyskinesias and psychosocial dysfunction (1–3). DDS occurs in Parkinson's Disease (PD) patients with a prevalence of about 8.8% (4). It is associated with younger age at onset of PD, male gender, impulsivity, and sensation-seeking personality traits, psychiatric history of depressive symptoms, and personal or family history of substance use (1–3). There are also reports of mania and hypomania associated with dopamine replacement therapy in PD with similar clinical factors associated with DDS and Impulsive Compulsive Behaviors (ICDs) (5).

We present a paradigmatic case of DDS, Mania, and Compulsive Buying in a 55-year-old male with PD.

## Case report

A 55-year-old male with PD was referred to our institution for psychiatric evaluation to undergo Deep Brain Stimulation (DBS) surgery due to debilitating dyskinesias and unpredictable and large periods of “OFF” states. He was diagnosed with PD at 50 years although the first symptoms were reported at age 43. Motor fluctuations and debilitating dyskinesias were reported 2 years before. He also had a history of Depressive Disorder since he was 41 years old. According to the information given by the patient and the family, there is no history in the family of knowing neurologic or psychiatric diseases. At admission, the patient presented dressed in colorful clothes and was wearing three gold necklaces. He presented an elated mood, disinhibition, logorrhea, an increase in speed of speech, increased self-esteem, diminished necessity to sleep, and paranoid delusions. He also displayed impulsive-compulsive behaviors namely compulsive buying and hoarding—he had bought over 5,000 pocket watches and 42 old and unusable cars, that he stored; he also stored old radio devices. He related these periods of excessive buying to periods of higher energy and higher doses of dopaminergic medication. At the time of this appointment, he was medicated with levodopa (total dose-−2,150 mg) and ropinirole 8 mg/daily corresponding to a levodopa equivalent daily dose (LEDD) of 2,310 mg. In a later appointment, he admitted to taking more levodopa pills than those prescribed by the doctor. He maintained the manic symptoms previously described. He also reported waking up in the middle of the night to take dopaminergic pills, associated with an increase in new activities and projects. The patient developed an interest in “old things” and new daily routines. He started leaving home early in the morning and buying old objects. Despite having no insight into these new behaviors, he was admitted voluntarily to the psychiatric ward. During the hospitalization, the dopaminergic medication was gradually reduced (LEDD-−2,030 mg), pill taking was controlled and he did not have access to extra pills. Valproic acid and quetiapine extended release were started and increased to 1,000 and 300 mg/daily, respectively and ropinirole was tapered off to 4 mg. The maniac symptoms gradually decreased and there was no worsening of Parkinson's motor symptoms or any change in dyskinesias. He was discharged 29 days after his admission to the psychiatry ward in clinical remission.

## Discussion

We provide a clinical description of a paradigmatic case of DDS, Mania, and Compulsive Buying in a patient with Parkinson's Disease. Knowledge about the phenomenology of DDS in patients with PD is still limited and reports of DDS comorbid with other ICDs and Mania are still scarce.

DDS is an uncommon condition, described for the first time by Giovannoni et al. (1). The estimated prevalence of 3.4–4% (1, 6). It is associated with other compulsive impulsive behaviors like *punding* and impulse control disorders. The use of high doses of levodopa and dopaminergic agonists with short-acting profiles are known risk factors.

The pathophysiology is not clear (7) but it probably occurs due to the loss of dopaminergic neurons in the *substantia nigra pars compacta* (SNc) and in the ventral tegmental area (VTA) with depletion of dopamine in the nigrostriatal motor pathway (2, 8). The dopaminergic stimulation of the *nucleus accumbens* (NAc) is essential to the reward effect of medication. The over-stimulation of the mesolimbic system can impact the development of rewarding behaviors (2, 8). The addictive properties of dopaminergic medication can be explained by the dysregulation of dopamine in the ventral striatum. The compulsive use and chronic stimulation by dopaminergic medication can lead to the hypersensitivity of D3 receptors (2, 9).

Neuroplasticity induced by dopaminergic replacement therapy in ventral and dorsal striatal systems and subsequent long-term disruptions of signaling in the basal ganglia can be associated to behavioral and motor complications of compulsive medication use in PD. Dopamine in nucleus accumbens may be responsible for certain rewards in terms of seeking food or sex as well as financial and verbal rewards. Sensitization of ventral striatal networks to dopaminergic replacement therapy may be analogous to the neuroplastic changes in the dorsal striatum which can contribute to the motor complications such as dyskinesias and repetitive motor acts. Moreover, striatal dopamine denervation in Parkinson's disease and cortically-mediated impairments in goal directed function may enhance sensitization to dopaminergic replacement therapy which seem to contribute to DDS development (10).

The core symptoms of this syndrome are the early self-adjustment of dopaminergic medication with the use of total doses superior to the need to control motor symptoms (1). Patients report good response to levodopa in an early phase, describing elated mood after each dose taken (11). There are also descriptions of impulsivity, agitation, irritability, and low tolerance to frustration (1). Usually, patients develop incapacitating dyskinesias associated with peak dose (1). Despite dyskinesias, patients continue to try to anticipate taking levodopa and increase the daily dose ingested (1, 2). Patients try to buy dopaminergic medication without a medical prescription and try to hide it from family members (1).

PD patients treated with dopamine agonists have more ICD, in comparison with those not treated with dopamine agonists. The use of pramipexole and ropinirole showed more risk for ICD because of their selectivity for D2-like receptors (D3 and D4 receptors), which are localized in the mesocorticolimbic system, explaining the risk of developing ICD. Levodopa, especially at higher dosages, was also related with ICD but to a lesser level than DA treatment (12). However, besides the risk of dopamine agonists, levodopa was more related with DDS than dopamine agonists, in contrary to what was observed in ICDs (13). Parkinson's disease patients with DDS exhibited enhanced levodopa-induced ventral striatal dopamine release compared with levodopa-treated patients with Parkinson's disease without DDS (14). PD patients with DDS show higher levodopa-induced ventral striatal dopamine release as compared to levodopa-treated patients with PD but who do not compulsively ingest dopaminergic drugs (15). Levodopa is believed to be the most potent trigger for the development of DDS (14).

Therapeutic management of DDS is complex and involves gradual tapering or discontinuation of dopaminergic medication, although discontinuation may be associated with severe motor disorders, dysphoria, irritability, depression, and anxiety (1, 2).

Some of the features respond to the minimization of short duration response or reduction of the dosage, while behavioral disturbances often require antipsychotic treatment with clozapine or quetiapine. Other authors previously described the efficacy of valproate in PD patients presenting with DDS, without observing the valproic acid-associated worsening of parkinsonism (15, 16). Valproate may reduce the substantia nigra reticulata output once maybe the excess dopaminergic medication drives increased activity in the ventral striatal reward pathway (17). Can also reduce the arachidonic acid cascade signaling pathway via D2-like receptors, modulating DDS behavior (15).

Mania and hypomania in PD are rarely reported besides the immediate post-DBS cases (5). Factors associated with the development of hypomania are similar to DDS and ICDs (higher LEDD, younger age of onset, and dyskinesias) (5). DDS is a significant clinical correlate of mania and hypomania, nevertheless, can exist independently (5). The pathophysiology is also identical to DDS, due to the sensitization of the ventral striatal dopaminergic receptors (5).

The effort in interrupting the processes of addiction may justify why limiting dopamine replacement therapy or changing to a non-pulsatile formulation can lead to these different symptoms. DBS, by acting in the subthalamic nucleus, can permit a reduction of dopamine replacement therapy and also attenuate the pulsatile nature. However, the use of DBS in resolving DDS has demonstrated ambiguous results, which may be related to the time taken from the start of PD to the use of DBS; less comorbidity, rapid withdrawal of levodopa post-DBS and use of dopamine agonists (13).

Increasing recognition of neuropsychiatric syndromes in PD patients is mandatory to improve treatment which frequently demands a specialized and multidisciplinary team.

## Data availability statement

The original contributions presented in the study are included in the article/supplementary material, further inquiries can be directed to the corresponding author.

## Ethics statement

Ethical review and approval was not required for the study on human participants in accordance with the local legislation and institutional requirements. Written informed consent from the patients/participants or patients/participants legal guardian/next of kin was not required to participate in this study in accordance with the national legislation and the institutional requirements. Written informed consent was obtained from the individual for the publication of any potentially identifiable images or data included in this article.

## Author contributions

CS: Writing – original draft, Writing – review & editing. MR: Writing – original draft, Writing – review & editing. IC: Writing – original draft, Writing – review & editing.

## References

[1] Giovannoni G O'Sullivan JD Turner K Manson AJ Lees AJ. Hedonistic homeostatic dysregulation in patients with Parkinson's disease on dopamine replacement therapies. J Neurol Neurosurg Psychiatry. (2000) 68:423–8. 10.1136/jnnp.68.4.423 10727476 PMC1736875

[2] Witjas T Eusebio A Fluchère F Azulay JP. Basal ganglia and limbic system: a new frontier - Addictive behaviors and Parkinson's disease. Reveu Neurologique. (2012) 168:624–33. 10.1016/j.neurol.2012.06.014 22921247

[3] Cilia R Siri C Canesi M Zecchinelli AL De Gaspari D Natuzzi F. Dopamine dysregulation syndrome in Parkinson's disease: from clinical neuropsychological characterization to management long-term outcome. J Neurol Neurosurg Psychiatry. (2014) 85:311–8. 10.1136/jnnp-2012-303988 23591553

[4] Barbosa P Djamshidian A Lees AJ Warner TT. The outcome of dopamine dysregulation syndrome in Parkinson's disease: a retrospective postmortem study. Mov Disord Clin Pract. (2018) 5:519–22. 10.1002/mdc3.12671 30515441 PMC6207120

[5] Maier F Merkl J Ellereit AL Lewis CJ Eggers C Pedrosa DJ. Hypomania and mania related to dopamine replacement therapy in Parkinson's disease. Parkinson Relat Disord. (2014) 20:421–7. 10.1016/j.parkreldis.2014.01.001 24467817

[6] Pezzella FR Colosimo C Vanacore N Di Rezze S Chianese M Fabbrini G. Prevalence and clinical features of hedonistic dysregulation syndrome in Parkinson's disease. Mov Disord. (2005) 20:77–81. 10.1002/mds.20288 15390130

[7] Kurlan R. Disabling repetitive behaviors in Parkinson's disease. Mov Disord. (2004) 19:433–7. 10.1002/mds.10625 15077241

[8] Rodriguez-Oroz MC Jahanshahi M Krack P Litvan I Macias R Bezard E. Initial clinical manifestations of Parkinson's disease: features and pathophysiological mechanisms. Lancet Neurol. (2009) 8:1128–39. 10.1016/S1474-4422(09)70293-5 19909911

[9] Berk M Dodd S Kauer-Sant'anna M Malhi GS Bourin M Kapczinski F. Dopamine dysregulation syndrome: implications for a dopamine hypothesis of bipolar disorder. Acta Psychiatr Scand. (2007) 116:41–9. 10.1111/j.1600-0447.2007.01058.x 17688462

[10] Evans AH Lees AJ. Dopamine dysregulation syndrome in Parkinson's disease. Curr Opin Neurol. (2004) 17:393–8. 10.1097/01.wco.0000137528.23126.41 15247533

[11] Borek LL. Levodopa addiction in idiopathic Parkinson's disease. Neurology. (2005) 65:1508. 10.1212/01.wnl.0000183147.59483.ab 16275855

[12] Weintraub D Claassen DO. Impulse control and related disorders in Parkinson's disease. Int Rev Neurobiol. (2017) 133:679–717. 10.1016/bs.irn.2017.04.006 28802938

[13] Warren N O'Gorman C Lehn A Siskind D. Dopamine dysregulation syndrome in Parkinson's disease: a systematic review of published cases. J Neurol Neurosurg Psychiatry. (2017) 88:1060–4. 10.1136/jnnp-2017-315985 29018160

[14] O'Sullivan SS Evans AH Lees AJ. Dopamine dysregulation syndrome: an overview of its epidemiology, mechanisms and management. CNS Drugs. (2009) 23:157–70. 10.2165/00023210-200923020-00005 19173374

[15] Sriram A Ward HE Hassan A Iyer S Foote KD Rodriguez RL. Valproate as a treatment for dopamine dysregulation syndrome (DDS) in Parkinson's disease. J Neurol. (2013) 260:521–7. 10.1007/s00415-012-6669-1 23007193

[16] Brugger F Bhatia KP Besag FM. Valproate-associated Parkinsonism: a critical review of the literature. CNS Drugs. (2016) 30:527–40. 10.1007/s40263-016-0341-8 27255404

[17] Evans AH Pavese N Lawrence AD Tai YF Appel S Doder M. Compulsive drug use linked to sensitized ventral striatal dopamine transmission. Ann Neurol. (2006) 59:852–8. 10.1002/ana.20822 16557571

